# The Impact of Digital Hospitals on Patient and Clinician Experience: Systematic Review and Qualitative Evidence Synthesis

**DOI:** 10.2196/47715

**Published:** 2024-03-11

**Authors:** Oliver J Canfell, Leanna Woods, Yasaman Meshkat, Jenna Krivit, Brinda Gunashanhar, Christine Slade, Andrew Burton-Jones, Clair Sullivan

**Affiliations:** 1 Centre for Health Services Research Faculty of Medicine The University of Queensland Brisbane Australia; 2 Queensland Digital Health Centre Faculty of Medicine The University of Queensland Brisbane Australia; 3 Digital Health Cooperative Research Centre Australian Government Sydney Australia; 4 UQ Business School Faculty of Business, Economics and Law The University of Queensland Brisbane Australia; 5 School of Clinical Medicine Faculty of Medicine The University of Queensland Brisbane Australia; 6 Institute for Teaching and Learning Innovation The University of Queensland Brisbane Australia; 7 Metro North Hospital and Health Service Department of Health Queensland Government Brisbane Australia

**Keywords:** electronic medical record, electronic health record, health care professionals, patients, patient satisfaction, hospitals, eHealth, attitude, perception, systematic, digital hospital, digital hospitals, experience, satisfaction

## Abstract

**Background:**

The digital transformation of health care is advancing rapidly. A well-accepted framework for health care improvement is the Quadruple Aim: improved clinician experience, improved patient experience, improved population health, and reduced health care costs. Hospitals are attempting to improve care by using digital technologies, but the effectiveness of these technologies is often only measured against cost and quality indicators, and less is known about the clinician and patient experience.

**Objective:**

This study aims to conduct a systematic review and qualitative evidence synthesis to assess the clinician and patient experience of digital hospitals.

**Methods:**

The PRISMA (Preferred Reporting Items for Systematic Reviews and Meta-Analyses) and ENTREQ (Enhancing the Transparency in Reporting the Synthesis of Qualitative Research) guidelines were followed. The PubMed, Embase, Scopus, CINAHL, and PsycINFO databases were searched from January 2010 to June 2022. Studies that explored multidisciplinary clinician or adult inpatient experiences of digital hospitals (with a full electronic medical record) were included. Study quality was assessed using the Mixed Methods Appraisal Tool. Data synthesis was performed narratively for quantitative studies. Qualitative evidence synthesis was performed via (1) automated machine learning text analytics using Leximancer (Leximancer Pty Ltd) and (2) researcher-led inductive synthesis to generate themes.

**Results:**

A total of 61 studies (n=39, 64% quantitative; n=15, 25% qualitative; and n=7, 11% mixed methods) were included. Most studies (55/61, 90%) investigated clinician experiences, whereas few (10/61, 16%) investigated patient experiences. The study populations ranged from 8 to 3610 clinicians, 11 to 34,425 patients, and 5 to 2836 hospitals. Quantitative outcomes indicated that clinicians had a positive overall satisfaction (17/24, 71% of the studies) with digital hospitals, and most studies (11/19, 58%) reported a positive sentiment toward usability. Data accessibility was reported positively, whereas adaptation, clinician-patient interaction, and workload burnout were reported negatively. The effects of digital hospitals on patient safety and clinicians’ ability to deliver patient care were mixed. The qualitative evidence synthesis of clinician experience studies (18/61, 30%) generated 7 themes: inefficient digital documentation, inconsistent data quality, disruptions to conventional health care relationships, acceptance, safety versus risk, reliance on hybrid (digital and paper) workflows, and patient data privacy. There was weak evidence of a positive association between digital hospitals and patient satisfaction scores.

**Conclusions:**

Clinicians’ experience of digital hospitals appears positive according to high-level indicators (eg, overall satisfaction and data accessibility), but the qualitative evidence synthesis revealed substantive tensions. There is insufficient evidence to draw a definitive conclusion on the patient experience within digital hospitals, but indications appear positive or agnostic. Future research must prioritize equitable investigation and definition of the digital clinician and patient experience to achieve the Quadruple Aim of health care.

## Introduction

### Background

Investment in digital health is advancing rapidly. In 2020, the total global funding for digital health was the highest recorded at US $26.5 billion [1]. A global appetite for digital health, fueled recently by the COVID-19 pandemic and the associated rapid adoption of point-of-care technological solutions [2], including telehealth [3], has driven the digital disruption of health care. A core pillar of digital health investment is the digital transformation of hospitals [4].

The World Health Organization Global Strategy on Digital Health (2020-2025) recommends the implementation of a national digital health architecture, including digital hospitals [5]. Digital hospitals represent significant jurisdictional investments to improve the quality and safety of acute care [6]. A digital hospital uses a comprehensive electronic medical record (EMR) to achieve its clinical goals [7] and is becoming the predominant method of care delivery worldwide. These new digital hospital environments radically disrupt well-rehearsed clinical workflows and create unfamiliar environments for patients and clinicians, potentially affecting quality, safety, and experience of care [8-10].

It has been difficult to determine the value of digital hospital implementations as what is considered valuable changes over time and place and from person to person [11]. Previous studies evaluating digital health implementations have focused on three domains: (1) improving patient or hospital outcomes using quantitative evaluation [12]; (2) exploring patient [13] and clinician behavior, workflows, and attitudes toward EMRs or digital hospital transformations [10]; and (3) quantifying value using economic evaluations [4]. Evidence to date demonstrates conflicting impacts of EMRs on hospital practice, with positive indications for medication safety, guideline adherence, and decision support [12,14] and negative indications for physician-patient communication, staff attitude, and workflow disruption [15,16]. Focusing on the narrow aspects of digital health implementations has resulted in patchy assessments of the value of digital health technologies.

**Figure 1:**
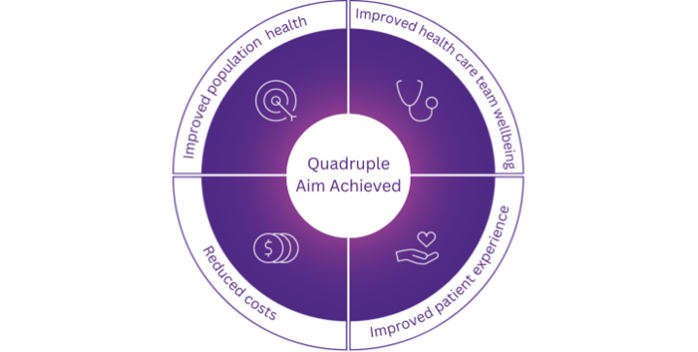
Quadruple Aim of health care [17].

The Quadruple Aim is the overarching goal of a learning health care system of enhancing patient experience, improving population health, reducing health care costs, and improving the provider experience (Figure 1) [17]. The Quadruple Aim of health care has been proposed as a strategic compass to guide digital health investment planning, decision-making, and evaluation [11] and has been used in the health care workforce [18,19], innovation implementation [20], and COVID-19 pandemic [21] contexts to identify current trends and research gaps.

The experience of patients and clinicians has yet to be explored as important contributors to the Quadruple Aim. Previous evaluations of clinician experience have focused on individual retrospective recalls of attitudes, perceptions of EMR implementation [10], and observational time and motion studies [22]. Existing patient experience research has focused on bespoke digital systems for patient use (eg, internet-based care technologies, web-based patient platforms, and mobile health apps) or emerging trends (eg, COVID-19 impacts, effects of specific technologies, research methods, and new technologies) [23,24]. Traditional evaluations of technology in health care are selective in the outcomes they measure, with an overwhelming focus on clinical outcomes and efficiency.

### Objectives

To address this research gap, our research question was as follows: *what is the clinician and patient experience of digital hospitals?* We hypothesized that clinicians and patients would report digital hospital experiences positively (eg, patient safety benefits because of digital safeguards [25]), negatively (eg, productivity loss because of documentation burden [15]), and ambivalently (eg, no observed impact of the digital environment on patient experience [10]). The study aim was to conduct a systematic literature review and qualitative evidence synthesis to assess the clinician and patient experience of digital hospitals.

## Methods

### Search Strategy and Identification of Included Articles

This review adhered to the PRISMA (Preferred Reporting Items for Systematic Reviews and Meta-Analyses) statement checklist [26] (Multimedia Appendix 1 [26]) and the ENTREQ (Enhancing the Transparency in Reporting the Synthesis of Qualitative Research) guidelines [27] (Multimedia Appendix 2). The protocol for this review was registered in PROSPERO (CRD42021258719).

The PubMed, Embase, Scopus, CINAHL, and PsycINFO databases were searched twice—on June 24, 2021 (version [V1]), and subsequently on June 23, 2022 (version [V2])—using the same search strategy restricted by year of publication (2010 to the present) because of the relative novelty of the digital transformation of acute care.

The search strategy was initially developed in PubMed using the population, intervention, comparison, and outcome structure (Table 1) [28] and translated to other databases (Multimedia Appendix 3). We define experience as an individual’s perception of events, incorporating themes of expectation and satisfaction. Thus, synonyms for “experience” were used to characterize the outcome. A combination of indexed terms (eg, Medical Subject Headings) and keywords identified after consultation with a research librarian and subject matter experts was used. Truncations, synonyms, and terminological variations (eg, “EMR” vs “EHR”) were also used.

**Table 1:** Final search strategy executed in PubMed (version 1: June 24, 2021; version 2: June 23, 2022).

PICO^a^ category	Domain	Search terms
Population	Patients and clinicians in a digital hospital	“Hospitals” (MeSH^b^) OR “hospital*” (tiab^c^)
Intervention	Digital hospital (using an EMR^d^ or EHR^e^)	“electronic health records” (MeSH) OR “electronic health record*”(ti^f^) OR “electronic medical record*” (ti) OR “emr” (ti) OR “EHR” (ti) OR “digital hospital*” (tiab) OR “smart hospital*” (tiab) OR “digital hospital*” (tiab) OR “computerized medical record” (tiab)
Comparison	None	None
Outcome	Experience	“satisfaction” (tiab) OR “experience” (tiab) OR “attitude*” (tiab) OR “perception*” (tiab) OR “opinion*” (tiab) OR “behavior*” (tiab) OR “behaviour*” (tiab)

^a^PICO: population, intervention, comparison, and outcome.

^b^MeSH: Medical Subject Headings.

^c^tiab: title and abstract.

^d^EMR: electronic medical record.

^e^EHR: electronic health record.

^f^ti: title.

In total, 2 reviewers (YM and OJC) performed title and abstract screening. Full-text review was then performed based on the eligibility criteria. Backward citation tracking (snowballing) was used to identify additional articles in the reference lists of included articles and relevant reviews. Decision conflicts were resolved through internal discussion or by involving a third reviewer’s opinion (C Slade) when required.

### Eligibility Criteria

Studies were included if they described a quantitative, qualitative, or mixed methods investigation of clinician or adult inpatient “experience” in a digital hospital (Table 2). This review focused on multidisciplinary clinicians and adult inpatients as a first step in synthesizing evidence of digital hospital experience. Pediatric inpatients were excluded because of possible environmental factors that could confound the digital hospital experience (eg, patient entertainment systems). Our study setting prioritized EMRs—a real-time patient health record that collects, stores, and displays clinical information in a tertiary setting [29] (eg, Cerner Millenium and Epic)—as the foundation of a digital hospital [30] as opposed to an electronic health record (or personal health record), which displays summarized patient information to the consumer in the community and across multiple health care providers [29] (eg, My Health Record [Australia], the National Health Service app [United Kingdom], and personal health record [Ministry of National Guard Health Affairs, Kingdom of Saudi Arabia]). The terms “EMR” and “EHR” may be used interchangeably in some countries, so both terms were adopted in the search strategy. The stage of digital hospital implementation (eg, EMR maturity [31]) was not considered.

**Table 2:** Study eligibility criteria for this systematic review and qualitative evidence synthesis.

Domain	Inclusion criteria	Exclusion criteria
Population	Multidisciplinary clinicians, including physicians, surgeons, nurses, allied health professionals and pharmacists, or adult inpatients	Nonclinical hospital staff, including hospital executive and administration staff Pediatric inpatients
Setting	Defined as a digital hospital or defined as a digital hospital by our research team—an acute care (hospital) service that uses an EMRa or any terminological variation to contribute to the goals of the health service [30]	Study not conducted in an acute hospital setting (eg, primary care, secondary care, internet-based health interventions, IoTb interventions, Hospital in the Home services, telehealth in primary care settings, mobile health, and public health) Study not concerning an EMR or any terminological variation in a hospital setting
Design	Qualitative methods for data collection (eg, interviews, focus groups, or ethnography) and data analysis (eg, thematic analysis or content analysis) Quantitative methods (eg, survey) to collect and analyze data Mixed methods studies in which either component fulfilled our eligibility criteria	Qualitative studies that do not include verbatim quotes from participants (to enable evidence synthesis) Articles not published as full-text empirical studies (ie, abstracts, conference proceedings, gray literature, dissertations, or theses; because of depth and quality variations) Narrative or systematic literature reviews; however, their reference lists were checked for relevant articles before exclusion (as our review focused on individual empirical studies) Articles published before 2010 (because of the rapid adoption of EMRs in hospitals since 2010) Articles not published in English (because of limited funding available for large-scale translation)

^a^EMR: electronic medical record.

^b^IoT: Internet of Things.

### Data Extraction

The Covidence software (Veritas Health Innovation) and Excel (Microsoft Corp) facilitated data extraction of study details (Multimedia Appendix 4). For studies eligible for the qualitative evidence synthesis (eg, presence of verbatim participant quotes), additional data that explained the qualitative findings were extracted, including the primary and main themes; secondary and subthemes; minor and unexpected themes; participant quotations; and any text labeled within the “results” or “findings” (ie, narrative) sections, including data-driven discoveries, judgments, or explanations the researchers offered about their phenomena [32,33]. All extracted data were cross-checked by a second reviewer for accuracy, and any discrepancies were resolved through discussion. Owing to the heterogeneity in study design, populations, and outcome measures for quantitative studies, a meta-analysis was inappropriate, and a narrative synthesis of quantitative study results was conducted.

### Quality Assessment

The quality of the included studies was assessed using the Mixed Methods Appraisal Tool (MMAT) [34]. Each study’s methodology was evaluated against 5 criteria (*Yes*, *No*, or *Can’t tell*) that differed between study designs (qualitative, quantitative randomized controlled trial, quantitative nonrandomized, quantitative descriptive, and mixed methods). The included studies were divided equally among 5 reviewers (LW, YM, C Slade, JK, and OJC). An MMAT star rating was generated for each article (*Yes*=1 star; up to 5 stars in total), a method adapted from a recent systematic review by Freire et al [35], and the scores were cross-checked by a second reviewer. Discrepancies were discussed and resolved within the research team. No study was excluded from the review based on its MMAT score.

### Data Synthesis

Data synthesis was conducted in 2 stages in accordance with our study aims.

#### Systematic Review: Narrative Synthesis

A narrative (qualitative) synthesis of quantitative studies was conducted to summarize and compare key findings [36]. We first developed a preliminary synthesis based on extracted data and then explored the relationships within and between studies to identify and explain any heterogeneity. Identified experience outcomes were inductively grouped together based on similarity (eg, ease of use and user-friendliness), and the group was given a descriptor (eg, usability) that would accurately reflect the experience outcomes within the group.

#### Qualitative Evidence Synthesis

The qualitative evidence synthesis [37] was conducted in 2 steps.

##### Step 1: Automated Text Analytics Using Machine Learning

Step 1 was undertaken using the text analytics tool *Leximancer* (version 4.5; Leximancer Pty Ltd) [38], an increasingly adopted [39] approach to qualitative analysis that is 74% effective at mapping complex concepts from matched qualitative data and >3 times faster than manual thematic analysis [40]. Leximancer applies an unsupervised machine learning algorithm and inbuilt thesaurus to uncover networks or patterns of word- and namelike terms in a body of text [41,42]. Leximancer then generates interconnections, structures, and patterns among terms to develop “concepts”—collections of words that are linked together within the text—and group them into “themes”—concepts that are highly connected. The interrelationships between concepts and themes are visualized on a map. Advantages include expediting the early stages of qualitative analysis and providing a first impression of meaning within qualitative data that limits researcher bias.

After data extraction, qualitative data from the included articles were synthesized into 3 data sets—“themes” (primary, secondary, and minor), “quotes” (from participants), and “narrative” (any text under the “results” or “discussion” sections)—ready for separate Leximancer analysis. We chose to analyze each data set separately to account for any significant (but unknown at the time) heterogeneity across the studies. In total, 3 researchers were allocated 1 data set each using Leximancer to create an initial concept map without altering any settings. Initial concepts were reviewed for meaning, and redundant conversational words were removed where appropriate (eg, *study*, *doing*, and *participants*). Concept variations of EMR or electronic health record were removed as this was labeled as the independent variable and the target context already under analysis. Concept variations (eg, *patient* and *patients*) were merged where necessary. All other software settings were kept as the default values.

##### Step 2: Researcher-Led Thematic Analysis

The preliminary themes and concepts identified via text analytics underwent validation and researcher-led thematic analysis in accordance with a modified version of the method by Thomas and Harden [33]. First, the top 5 Leximancer-identified concepts (eg, “patient”) were identified and connected with their 2 most related concepts (eg, “patient” AND “documentation” or “patient” AND “time”) to create a concept grouping. In total, 3 researchers (OJC, LW, and JK) validated each Leximancer concept grouping by extracting relevant text and generating a preliminary interpretation of the meaning of each concept grouping, which was cross-checked between researchers. Researchers (OJC, LW, JK, and BG) then worked collaboratively across all 3 data sets to conduct a rapid thematic analysis using a cluster and name technique to generate a working thematic framework [43]. Through an iterative and interpretive process, researchers then grouped similar concepts into parent themes. Discrepancies were resolved through discussion until the final themes were decided and approved by consensus.

## Results

### Identification of Included Articles

**Figure 2:**
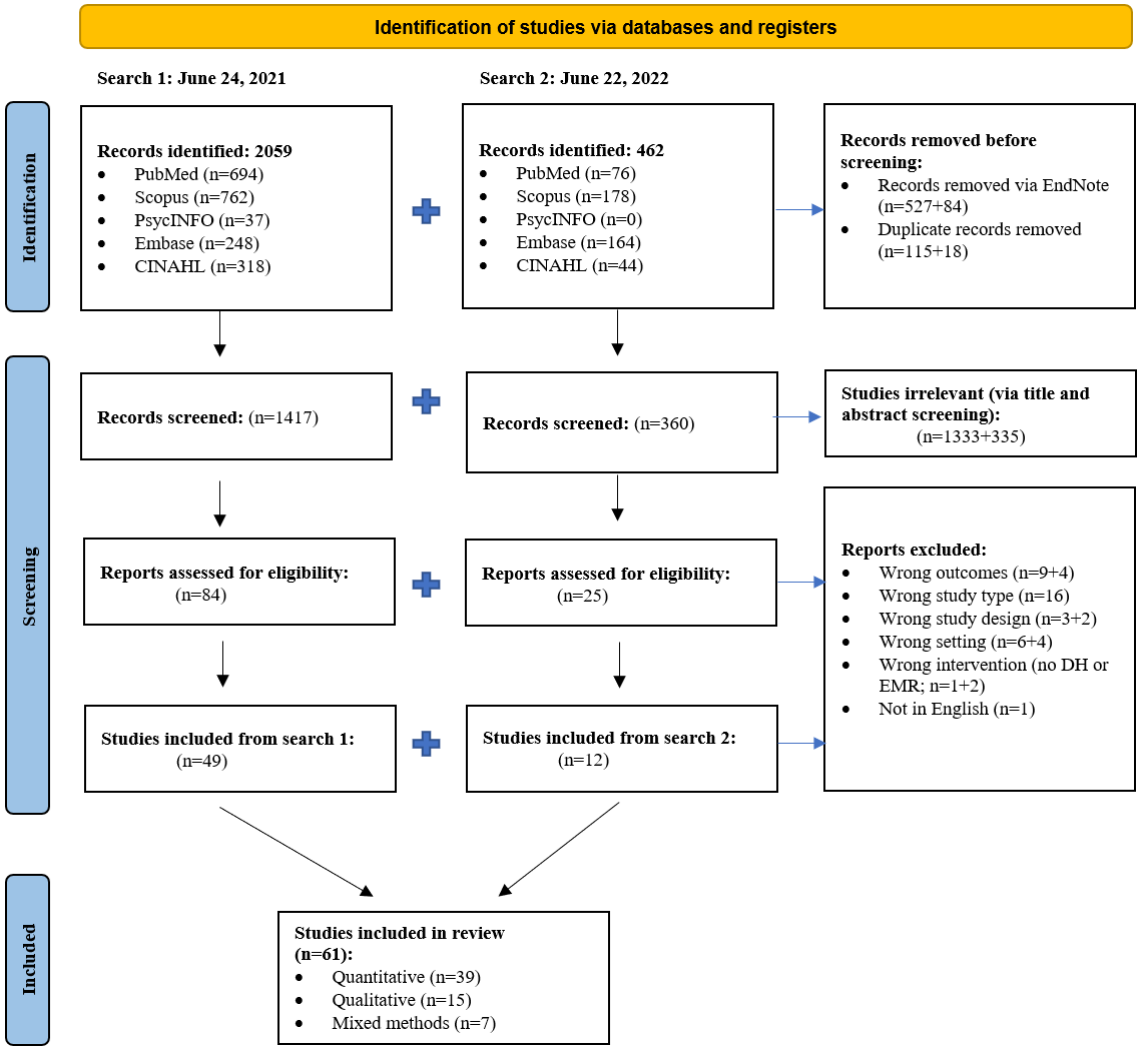
PRISMA (Preferred Reporting Items for Systematic Reviews and Meta-Analyses) flow diagram for the systematic review and qualitative evidence synthesis. DH: digital hospital; EMR: electronic medical record.

In total, 2059 studies were identified from the first search (V1), and an additional 462 studies were identified from the second search (V2; Figure 2). Following duplicate removal and title and abstract screening, a total of 109 studies (V1: n=84, 77.1%; V2: n=25, 22.9%) remained and underwent full-text review. Of these 109 studies, 61 (56%) met our inclusion criteria and were included in this review, comprising quantitative (n=39, 64%), qualitative (n=15, 25%), and mixed methods (n=7, 11%) designs.

### Quality Assessment

In total, 52% (32/61) of the included studies met all 5 MMAT quality criteria (Multimedia Appendix 5 [44-104]). An additional 20% (12/61) of the studies met 4 out of 5 of the quality criteria. Only 7% (4/61) of the studies met ≤2 of the 5 quality criteria, of which 75% (3/4) were mixed methods studies and 25% (1/4) had a quantitative descriptive design. For these studies, a score of ≤2 indicated inadequate sampling, and in the case of the mixed methods studies, the integration of and inconsistencies between quantitative and qualitative elements were not adequately described.

### Characteristics of the Included Studies

#### Study Design

Of the 61 included articles (Multimedia Appendix 6 [44-104]), most (n=39, 64%) adopted quantitative methods to assess clinician and patient experiences. Most quantitative studies (31/39, 79%) conducted descriptive cross-sectional surveys to assess experience at one point. A total of 21% (8/39) of the studies assessed clinician and patient experience through quantitative nonrandomized methods in the pre– and post–EMR implementation periods. A minority of the included studies (15/61, 25%) qualitatively assessed experience through interviews, focus groups, and ethnographic observations. A total of 11% (7/61) of the studies used both quantitative and qualitative methods (mixed methods).

#### Setting

The most common country of study was the United States (21/61, 34%), followed by Australia (6/61, 10%), Saudi Arabia (5/61, 8%), and Canada (4/61, 7%). More than half (32/61, 52%) of the included studies were published after 2018. The settings included diversity across large tertiary academic hospitals and private hospitals in rural and metropolitan settings. The number of participating hospitals ranged from 1 to 2836.

#### Participants

In total, 90% (55/61) of the studies investigated the clinician experience using quantitative, qualitative, or mixed methods. Within the clinician experience group, 35% (19/55) of the studies included all EMR users, followed by nursing staff only (17/55, 31%) and physicians only (15/55, 27%). Study participation ranged from 8 to 3610 across the clinician experience studies. Only 16% (10/61) of the included studies investigated the patient experience. In total, 60% (6/10) of the studies focused exclusively on the patient experience with EMR, and 40% (4/10) included perspectives from both stakeholder groups. Patient participant counts ranged from 11 to 34,425.

### Quantitative Results: Clinician and Patient Experience in Digital Hospitals

#### Overview

Table 3 reports the outcome measures of the digital hospital experience identified in the studies with quantitative components (46/61, 75%; 39/46, 85% quantitative and 7/46, 15% mixed methods).

**Table 3:** Measures of the digital hospital experience identified in the included studies (quantitative and mixed methods studies; n=46).

Experience outcome		Studies, n (%)	References
**Patient experience (n=9; n=7 quantitative and n=2 mixed methods)**			
	Patient satisfaction	6 (67)	Kazley et al [48] Hu et al [84] Jarvis et al [51] Monturo et al [102] Tian et al [96] Burridge et al [69]
	Recommended the hospital	2 (22)	Kazley et al [48] Hu et al [84]
	Good discharge information	3 (33)	Kazley et al [48] Hu et al [84] Burridge et al [69]
	Provider-patient interaction (patient perspective)	3 (33)	Migdal et al [57] Monturo et al [102] Burridge et al [69]
**Clinician experience (n=41; n=34 quantitative and n=7 mixed methods)**			
	Overall EMR^a^ satisfaction	24 (59)	Tajirian et al [88] Schwarz et al [87] Schopf et al [77] Williams et al [79] Alobo et al [80] Bani‐issa et al [62] Alharthi et al [54] Harmon et al [59] Kaipio et al [85] Kutney-Lee et al [76] Lakbala and Dindarloo [56] Tilahun and Fritz [61] Tubaishat [72] Abu Raddaha [70] Top and Gider [49] Alsohime et al [73] Al-Mujaini et al [46] Shaker et al [60] Claret et al [47] Al Otaybi et al [98] Cho et al [89] Czernik et al [101] Jedwab et al [97] Welchen et al [104]
	Usability	19 (46)	De Groot et al [82] Schwarz et al [87] Schopf et al [77] Aldosari et al [68] Eden et al [83] Alharthi et al [54] Top et al [53] Kutney-Lee et al [76] Bossen et al [50] Kaipio et al [66] Tubaishat [72] Abu Raddaha [70] Top and Gider [49] Strudwick et al [71] Arikan et al [99] Al Otaybi et al [98] Cho et al [89] Lloyd et al [93] Welchen et al [104]
	Adaptation	14 (34)	Schopf et al [77] Schenk et al [64] Hung et al [75] Lakbala and Dindarloo [56] Bossen et al [50] Kaipio et al [66] Abu Raddaha [70] Strudwick et al [71] Shaker et al [60] Claret et al [47] Arikan et al [99] Al Otaybi et al [98] Czernik et al [101] Lloyd et al [93]
	Data accessibility	18 (44)	De Groot et al [82] Schwarz et al [87] Aldosari et al [68] Alobo et al [80] Eden et al [83] Alharthi et al [54] Top and Gider [49] Hung et al [75] Kutney-Lee et al [76] Bossen et al [50] Kaipio et al [66] Abu Raddaha [70] Top et al [53] Strudwick et al [71] Arikan et al [99] Al Otaybi et al [98] Lloyd et al [93] Welchen et al [104]
	Provider-patient interaction (clinician perspective)	3 (7)	Ratanawongsa et al [67] Al Otaybi et al [98] Czernik et al [101]
	Workload and burnout	19 (46)	Tajirian et al [88] Schopf et al [77] Kutney-Lee et al [92] Alobo et al [80] Eden et al [83] Schenk et al [64] Harmon et al [59] Lakbala and Dindarloo [56] Tilahun and Fritz [61] Bossen et al [50] Strudwick et al [71] Shaker et al [60] Claret et al [47] Arikan et al [99] Al Otaybi et al [98] Czernik et al [101] Heponiemi et al [90] Jedwab et al [97] Luyten and Marneffe [94]
	Patient safety	14 (34)	Tajirian et al [88] Schwarz et al [87] Williams et al [79] Alobo et al [80] Top et al [53] Hung et al [75] Kaipio et al [85] Lakbala and Dindarloo [56] Bossen et al [50] Kaipio et al [66] Alsohime et al [73] Al Otaybi et al [98] Lloyd et al [93] Luyten and Marneffe [94]
	Delivery of care	12 (29)	De Groot et al [82] Top et al [53] Harmon et al [59] Kutney-Lee et al [76] Tilahun and Fritz [61] Abu Raddaha [70] Top and Gider [49] Alsohime et al [73] Al-Mujaini et al [46] Claret et al [47] Al Otaybi et al [98] Czernik et al [101]

^a^EMR: electronic medical record.

#### Patient Experience in a Digital Hospital

Of the 9 quantitative or mixed methods studies reporting the patient perspective, 7 (78%) [48,51,57,69,84,96,102] used different survey methods (eg, the Hospital Consumer Assessment of Healthcare Providers and Systems [HCAHPS] survey) to quantify “patient experience” using various satisfaction metrics. Of these studies, 57% (4/7) reported a positive association [48,57,69,84] between EMR and patient satisfaction scores and 43% (3/7) reported no substantial change [51,96,102]. In total, 22% (2/9) of the studies [67,92] used hospital outcomes rather than patient feedback, which did not meet our definition of “experience.”

A total of 33% (3/9) of the studies [57,96,102] reported patient experience before and after EMR implementation (or transition between EMR systems). Tian et al [96] surveyed 34,425 patients using the standardized HCAHPS survey and found a significant decreasing trend in patient experience scores for the 6 months after implementation followed by a return to baseline, with no significant changes overall. Monturo et al [102] surveyed 55 patients and found no significant changes in overall patient satisfaction.

Of the 9 studies, 3 (33%) cross-sectional studies [48,51,84] compared patient experiences in hospitals with and without an advanced EMR. Hu et al [84], using the HCAHPS survey at 1006 hospitals, and Kazley et al [48], using Hospital Compare data at 2836 hospitals, both found a positive association between EMR adoption and overall hospital rating and discharge information. Jarvis et al [51] found no significant difference in HCAHPS scores in advanced EMR versus non–advanced EMR hospitals.

#### Clinician Satisfaction With the EMR

Of the 41 quantitative studies that investigated the clinician experience, 24 (59%) included an *overall EMR satisfaction* metric, and 71% (17/24) of these studies reported a positive sentiment [47,49,59,60,62,70,72,73,76,79,80,85,87-89,98,101]. For instance, Kutney-Lee et al [76] used the registered nurse forecasting study (RN4CAST-US) nursing survey with 12,377 nurses across 353 hospitals and found a 74.9% “satisfaction with current EMR.” In total, 25% (6/24) of the studies [64,68,72,75,89,94] used features of the technology acceptance model, and “perceived usefulness” or “perceived value” was equated to *overall satisfaction*. Evidence of increasing satisfaction with increased digitization was found in a study that stratified results by level of EMR adoption, including groups for basic EMR (71.3% satisfaction reported) and comprehensive EMR (78.4% satisfaction) [76].

Of the 24 studies that reported *overall EMR satisfaction* as an outcome of clinician experience, 7 (29%) [46,54,56,61,77,97,104] reported negative sentiment with the EMR. For instance, Tilahun and Fritz [61] surveyed 406 clinicians and found that 64.4% were dissatisfied with the use of the EMR system; however, only 22.8% strongly disagreed with the following statement: “I prefer EMR than the paper record.” One study found that only 15.6% of respondents (n=141 physicians) felt that the EMR was an “effective tool” [46], and another found that only 38.9% of users (n=262 nurses and physicians using the National Usability-Focused Health Information System Scale) rated the EMR system as “high quality” [104].

#### Usability of the EMR by Clinicians

Of the 41 studies that included the EMR user perspective, 19 (46%) reported a *usability* metric, and 11 (58%) of these reported a positive sentiment [50,54,68,70,71,72,76,82,83,89,99]. Survey components that investigated usability used outcomes such as “ease of use,” “user friendly,” and “technical quality.”

Positive sentiment was considered to be >3.5/5 on a 5-point Likert scale or >50% agreement with usability statements. Of those using a Likert scale, statements such as “the health record I am working with is user-friendly” scored 3.62 (n=667 nurses) [82], and “perceived ease of use” scored 3.7 (n=1539 nurses in 15 hospitals) [72] and 3.78 (n=223 nurses) [89]. Aldosari et al [68] surveyed 153 nurses and found a 79.7% agreement with the following statement—“It is easy to use the EMR”—and 70.5% agreement with the following statement: “I find the EMR system interface to be user friendly.”

A negative sentiment regarding usability was found in 42% (8/19) of the studies [49,53,66,77,87,93,98,104]. In the study using the National Usability-Focused Health Information System Scale (n=3013 physicians), most participants (60.15%) disagreed with the following statement: “routine tasks can be performed in a straightforward manner without the need for extra steps using the systems” [66]. Comparatively fewer nurses (n=3560) in the same study disagreed with this statement [66].

#### Adaptation to New Systems

A total of 30% (14/41) of the studies discussed the experience of adapting existing workflows to integrate the new digital interface and transitioning to a digital environment on the wards [47,50,56,60,64,66,70,71,75,77,93,98,99,101]. Generally, the *adaptation* outcome had a negative sentiment from EMR users. One survey of 285 nurses 8 to 13 months after EMR implementation found that users felt that the EMR provided a “holistic view of the patient, but fragmentation and complexity introduce workflow challenges” [64]. Another study found that 35.1% of physicians (n=317) agreed with the following statement: “EMR does not disrupt workflow” [60]. A third study found that 48.7% of physicians (n=3013) and 62.3% of nurses (n=3560) disagreed with the following statement: “learning the EHR did not require a lot of training” [66].

#### Data Accessibility and Clinician-Patient Interaction

Data accessibility in digital hospitals was reported in 39% (18/41) of the quantitative studies [49,50,53,54,66,68,70,71,75,76,80,82,83,87,93,98,99,104]. Much of the sentiment was positive as clinicians agreed that the EMR allowed users to access information when and where they needed it. One survey of 153 nurses in a Saudi Arabian hospital found that 85.6% of respondents agreed with the following statement—“I have access to the information where I need it”—and 83.6% agreed with the following statement: “I have access to the information when I need it” [68]. One large cross-sectional study of 2684 clinicians found a 50.3% agreement with the following statement: “EMR provides precise information I need” [98]. Neutral sentiment was indicated in one study by an average of 3.5 (5-point Likert scale) in response to the following statement—“it is easy to find the information I need”—from 244 clinicians 2 months after EMR implementation [50]. The survey by Lloyd et al [93] found that 49% of physicians (n=224) and 59.4% of nurses (n=72) agreed that “it is easy to obtain necessary patient information using the EMR system.”

A total of 7% (3/41) [67,98,101] of the studies reported on the impact of EMR on clinician-patient interaction, with all 3 studies agreeing that the EMR reduced this communication. Czernik et al [101] found that 39% of 126 physicians agreed (7-point Likert scale) with the statement that EMR causes “lack of proper patient-doctor communication.” A total of 43% (3/7) [57,69,102] of patient experience studies reported on the impact of EMR on patient-provider interaction. Migdal et al [57] focused more specifically on physician-patient communication with their patient participants using a CICARE survey (17-question Likert scale) designed by the University of California, Los Angeles, Health system to assess resident physician performance. Of the 3417 patient surveys, Migdal et al [57] found that 9 of 16 relevant questions had statistically significant improvements after EMR implementation, suggesting improvement in communication between patients and providers after EMR implementation.

#### Workload and Burnout

Many studies (19/41, 46%) reported on the impact of EMR on clinical workload, including symptoms of burnout and subjective productivity. One cross-sectional study in Canada surveyed 208 physicians and found that 68.2% of respondents felt that the EMR “added to daily frustration”; 24.5% of respondents had one or more symptoms of burnout; and, of those with burnout, nearly 75% “identified EMR as contributor to burnout symptoms” [88]. Another study across 343 hospitals including 12,004 nurses compared EMR usability (as per the RN4CAST-US usability survey) with symptoms of burnout and found that lower EMR usability scores were associated with higher odds of burnout (odds ratio 1.41, 95% CI 1.21-1.64) [92]. Often, studies assessed workload in terms of productivity. In 5 low-resource hospitals 3 years after EMR implementation (n=405 physicians and nurses), 82.4% of physicians disagreed that “EMR improves productivity,” whereas 61% of nurses agreed [61].

#### Patient Safety and Delivery of Care

There was a mixed sentiment across 34% (14/41) of the quantitative studies [50,53,56,66,73,75,79,80,85,87,88,93,94,98], which included survey items on the impact of the EMR on patient safety. In total, 43% (6/14) of these studies [53,56,66,73,93,98] included survey items about EMR preventing errors in patient care, especially mistakes associated with medications. One study in a large specialist hospital in Nigeria (n=35 health care workers) found that the EMR made clinicians “more prone to errors” [80]. Similarly, Kaipio et al [66] found that less than half of the surveyed physicians (44.7%) and nurses (40.2%) agreed with the following statement: “IT systems help in preventing errors and mistakes associated with medication.” Conversely, Al Otaybi et al [98] (n=2684 health care workers) found that only 15.5% agreed with the following statement: “EMR increases the risk of making errors.” One study investigated the change in user experience over time and found that agreement with the statement that the EMR “improves prevention in errors and mistakes associated with medications” increased by 13% from 2010 to 2014 [85].

There was also a mixed sentiment across 29% (12/41) of the studies, which included outcomes on the impact of the EMR on clinicians’ ability to deliver care to their patients. In a large study of >12,000 US nurses, more than half (55.4%) reported agreement with the statement that the EMR “systems interfere with the provision of care” [76], and in a smaller study, 84.2% of participants disagreed with the statement that the EMR “system has positive impact on quality of care” [61]. Conversely, in the Netherlands, nurses were more likely to agree with the following statement: “the information in the health records supports my activities during the provision of care” [82].

### Qualitative Results: Clinician and Patient Experience in Digital Hospitals

A total of 18 studies (n=14, 78% qualitative and n=4, 22% mixed methods) with qualitative components were included in the qualitative evidence synthesis. Only 7% (1/15) of the qualitative studies in our review explored the patient experience in a digital hospital [52]; however, this study was excluded from the qualitative evidence synthesis and is reported narratively in the following paragraph. A total of 29% (2/7) of the mixed methods studies were also excluded for lacking direct participant quotes as per the exclusion criteria [50,75]; however, the quantitative results are reported in the previous sections.

Strauss [52] interviewed 11 patients about the dynamics with their nurses and the EMR. Similar to the qualitative evidence synthesis, participants described a positive perception of the EMR when the nurses acknowledged the participants before using the electronic device; however, many “expressed concerns [for] the privacy of their health record information.” Interestingly, participants’ expectations of the “clinical knowledge and competency of the nurse, within the technological arena, have increased with the implementation of the [EMR].”

### Qualitative Evidence Synthesis: Clinician Experience Only

#### Step 1: Automated Text Analytics Using Leximancer

Multimedia Appendix 7 presents the results of the automated text analytics using Leximancer. Figures S1 to S3 in Multimedia Appendix 7 present the intertopic concept maps derived from the themes, quotes, and narrative qualitative data, respectively.

Table S1 in Multimedia Appendix 7 compares the top 5 concepts and their 2 most related concepts identified by Leximancer across each qualitative group. Owing to the relative homogeneity in the top 5 concepts identified among the themes, quotes, and narrative qualitative data, it was decided to perform researcher-led thematic analysis (step 2) collectively instead of individually for each data group. This decision was not a predetermined method and was made organically during data analysis.

#### Step 2: Researcher-Led Thematic Analysis

##### Overview

Textbox 1 presents 7 themes that describe the clinician experience in digital hospitals derived from the qualitative evidence synthesis (18/61, 30% of the studies).

**Final themes describing the clinician experience in digital hospitals from the qualitative evidence synthesis (n=18).**

**Theme 1**

- Slow and inefficient digital documentation detracts from other clinical priorities.

**Theme 2**

- Inconsistent data quality and discoverability challenge clinician trust in making data-driven decisions.

**Theme 3**

- Digital technology creates new tensions that disrupt conventional health care relationships.

**Theme 4**

- Acceptance of digital hospitals is a value-based spectrum that changes over time.

**Theme 5**

- Clinicians value patient safety benefits while acknowledging concerns about new digital risks.

**Theme 6**

- Clinicians feel reliant on hybrid (digital and paper) workflows to maintain the standard of care.

**Theme 7**

- Clinicians worry about compromising patient data privacy to improve care efficiency.

##### Theme 1: Slow and Inefficient Digital Documentation Detracts From Other Clinical Priorities

Documentation was a time burden for clinicians with a slow and inefficient workflow [95] in which it was difficult to find information [95], there were “too many steps to accomplish simple tasks” [44], and users were required to re-enter the same data repeatedly [63].

Clinicians felt challenged by the requirement for accurate and complete documentation during the provision of patient care. Staff reported wanting to provide care but needing to complete medical records:

A lot of the time we’re having to say [to patients], “Oh look, I’ll have to come back to you, I’ve got to do my documentation.” [81]

The study by Schenk et al [64] concluded that “EHR implementation was disruptive to nursing care and adversely influenced nursing attitudes,” reporting “too many steps to find and chart information, information that is fragmented and overly complex, leading to workflow challenges and interruptions of care.” Shortcuts and quick orders save time [95] and were used as workarounds to improve efficiency.

##### Theme 2: Inconsistent Data Quality and Discoverability Challenge Trust in Decisions

Clinicians found that data quality improved in digital hospitals. Studies highlighted various benefits of digitization, including improved documentation of data [78], efficient display of information [95], improved data completeness [78], and improved documentation readability [63]. This was summarized by the following participant quote:

...the availability of data in the EHR is a good thing. [101]

“Note bloat” was reported as a theme regarding difficulties finding information in the digital system [95]. Discoverability challenged clinicians and negatively affected their trust in making data-driven decisions. It was difficult to find information [95] and easy to miss information [95], as described by one participant:

...you’ve got to look through 100 documents to find the information you are looking for. [65]

Inefficiencies in the EMR design may lead to inappropriate care:

A wrong decision happens based on the missing information. [45]

##### Theme 3: Digital Technology Creates New Tensions That Disrupt Conventional Health Care Relationships

Reduced patient contact [55] from EMRs “inserted” between the patient and clinician deteriorated the personal relationship [44]. There was the potential to lose focus on the patient, undermine rapport [86], and communicate with the computer in lieu of direct bedside patient communication [81]. The effect of digital documentation on trust in the psychiatrist-patient relationship was noted, and required open communication between the psychiatrist and patient to promote transparency about what was documented [100].

Managing disrupted communication [81] to preserve the patient’s personhood in the digital environment [81] created notable tension [69]. For example, the EMR can improve information accuracy [44] by viewing a patient history [103] or using a computer to facilitate conversation [86]. Preparing notes before consultations, minimizing screen use and explaining computer use, taking paper notes, sharing screens with patients, and viewing results digitally together “seems to counterbalance the negative effects of computer use” [86].

Interprofessional communication between clinicians was affected by digital notes “only when the data entered by different roles in the healthcare system are accurate, the clinicians can make timely and correct decisions” [103].

##### Theme 4: Acceptance of Digital Hospitals Is a Value-Based Spectrum That Changes Over Time

Individual beliefs about digital hospitals trended negatively (eg, threats to patient safety, waste of human resources, and perceived inefficiency), but objective experiences trended positively (eg, access to records, optimized treatment, efficiency and health system coordination [45], cost savings, improved productivity, and quality of care). Acceptance fluctuated between perception and reality—a mismatch between “work as imagined” and “work as done.”

Negative experiences with “work as imagined” were related to perceived inefficiencies [45]. Digital hospitals created their own unique time pressures with improved productivity in some cases [45], with participants viewing technology as “both time saving and time consuming” [74].

Positive experiences with “work as done” coincided with a longer time since implementation, when adoption had progressed, disruption to their work processes had eased, and workflows had been integrated [58]. Initially, clinicians reported having a negative first impression of the EMR, especially perceived complexity and ease of use [91].

##### Theme 5: Clinicians Value Patient Safety Benefits While Acknowledging Concerns About New Digital Risks

Digital hospitals generate patient safety benefits while creating new ways to make errors and increase risks. New errors may negatively affect patient safety. These include wrong patient errors, alert fatigue, inappropriate alerts, data entry errors, technical problems [78], field auto-population or auto-refresh errors, and the absence of aids for dose calculations [95]. Concerns regarding clinician overreliance on the system, ignoring correct alerts, and prioritizing system compliance over clinical accuracy were raised in some cases [45].

Reduced medication errors were the primary reported patient safety benefit, including awareness of known adverse reactions to medications [103] and improved legibility [78]. Clinical decision support enabled by the EMR was seen as a safety benefit to alert staff for prompt intervention [78]:

There is a pop up in the system which questions are you supposed to give that medication right now? [103]

The safety benefits of sharing medical information [100] and applying regulatory frameworks to the workflow [100] were noted. Entering clinical data into structured mandatory fields and managerial-level review improved thoroughness and was considered to enhance patient safety [78].

##### Theme 6: Clinicians Feel Reliant on Hybrid (Digital and Paper) Workflows to Maintain Standard of Care

Digital transformation caused workflow disruption. New digital workflows were time consuming, with a one-size-only user interface and limited ability to adapt to individual patient characteristics or change information once documented [86]. The digital interface was insufficient to meet clinical workflow needs. Often, EMR workflows were supplemented with paper workflows [81]. Paper enabled total customization to fit with workflow conventions and was a “cognitive support” to supplement personal workflows to plan and prioritize in a flexible, convenient, comfortable, and trusted way [58]:

I go to my [paper] notes and I make little boxes, and if I do those tasks I tick them. [81]

##### Theme 7: Clinicians Worry About Compromising Patient Data Privacy to Improve Care Efficiency

The improved documentation captured by the EMR created a perceived privacy risk for the patient. Perceptions on patient preferences to protect the disclosure of medical information involved elements such as diagnoses [100], mental health–related stigma [100], and patient distrust of the EMR system or its users [86]. Reported strategies enlisted by health care professionals were to only include clinical documentation that was general in nature, avoid labeling (eg, “mood disorder” instead of “depression”) [100], prioritize based on clinical relevance [100], or limit types of information to critical information related to medication that is considered essential knowledge [86].

## Discussion

### Principal Findings and Comparison With Prior Work

**Figure 3:**
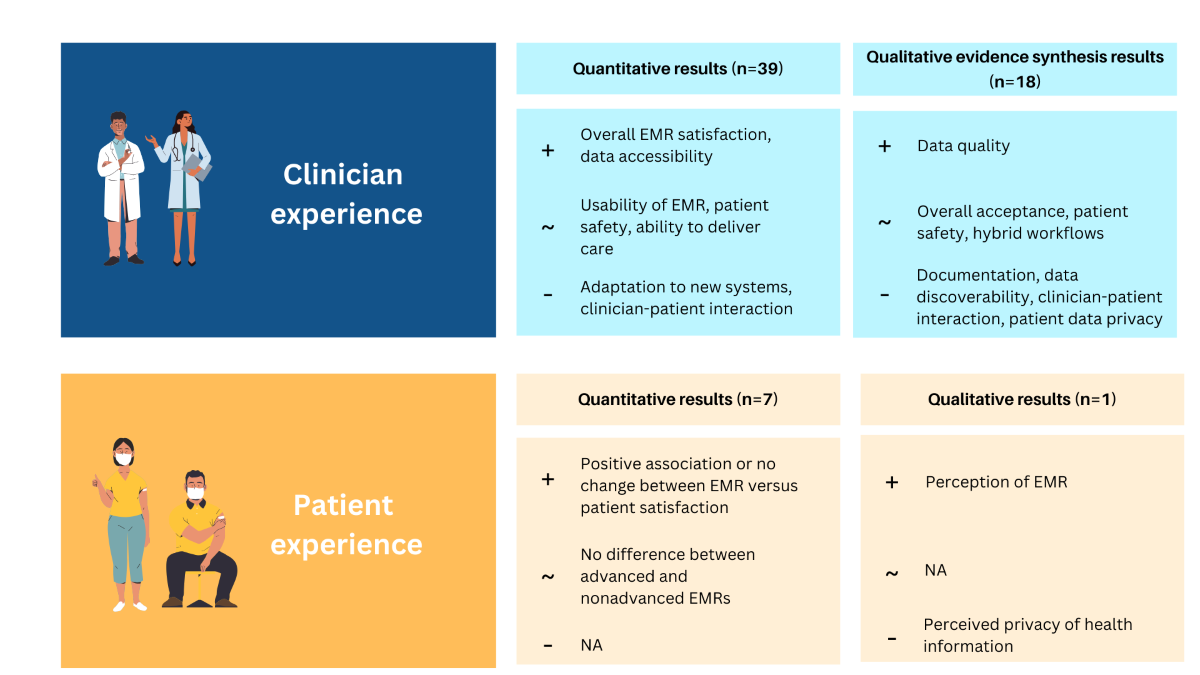
Summary of key findings on the clinician and patient experience of digital hospitals. +: positive; ~: mixed; −: negative; EMR: electronic medical record; N/A: not available.

Our findings revealed mixed and complex clinician and patient experiences of digital hospitals (Figure 3). Generally, clinicians reported positive overall satisfaction (17/24, 71% of the studies) with digital hospitals in quantitative measures; however, there were negative experiences for clinicians reported in qualitative studies, including compromised clinician-patient interactions, inefficient data workflows, and patient data privacy concerns. For example, acceptance of digital hospitals fluctuated over time and trended negatively if grounded in individual perceptions and beliefs (ie, “work as imagined”) yet trended positively if based on objective measures of practice (ie, “work as done”) [105]. These inconsistencies are likely reflective of the various contextual factors that influence experience, such as intervention design or stage of implementation. It is likely that the quantitative finding of mixed usability of EMRs explains clinician reliance on hybrid (digital and paper) workflows revealed in the qualitative evidence synthesis as clinicians seek to maintain their clinical workflow standard and validate data using additional sources of truth, such as paper [10].

The effect of digital hospitals on clinician-reported patient safety and clinician ability to deliver care were mixed; there was acknowledgment that digitization primarily reduces medication error risk but creates new risks driven by questionable data quality. In the small proportion of studies that explored patient experience (10/61, 16%), there was weak evidence supporting a positive association between digital hospitals and patient satisfaction scores. To our knowledge, this is the first review to systematically evaluate clinician and patient experience in digital hospitals and use qualitative evidence synthesis with machine learning (Leximancer) to consolidate identified themes in previous qualitative research into an empirical “umbrella” view of digital hospital experience.

Previous studies have evaluated the adoption of health ITs and identified enablers of and barriers to the routine use of EMRs in practice. The perceived value of the EMR to clinical workflows and data accessibility are key adoption facilitators, whereas cost and time consumption are barriers to adoption [106]. Our findings revealed that clinicians reported high satisfaction with digital hospitals and positively viewed data accessibility in quantitative measures; however, our qualitative evidence synthesis revealed themes of frustration with slow digital workflows and inconsistent data discoverability. Evidence of EMR adoption in low-income countries also highlights clinician perception of the EMR as a key facilitator and interoperability and clinician burnout as barriers, similar to our findings on the impact of EMR on workload and burnout symptoms [107].

Our review found that the patient experience of digital hospitals was reported disproportionately less frequently than the clinician experience. Evidence of patient satisfaction with EMRs was systematically reviewed in 2013, and it was found that a small number of studies (n=8) indicated positive patient satisfaction with the EMR in mixed settings across primary care, emergency, and outpatient departments [13]. This evidence is consistent with our findings of a positive or neutral association but remains grounded in cross-sectional methods that warrant rigorous trial evaluation. Beyond the clinician-facing EMR, patient-centered digital health records have emerged as a mechanism to engage and empower consumers living with chronic conditions. Patient portals within EMRs can contribute positively to health care quality and safety by improving medication adherence and clinician-patient communication [24] and have been shown to improve patient care navigation and disease knowledge without adverse effects and with high patient satisfaction [108]. Measuring satisfaction with digital hospitals using a simple quantitative scale is unlikely to capture the complexity and heterogeneity of digital hospital environments. There was dissonance in clinician perspective on data accessibility (or discoverability) between quantitative and qualitative studies. Clinicians reported objective satisfaction with data accessibility and positive attitudes toward data quality; however, they were dissatisfied with the inefficient workflows required to generate high-quality data (ie, input) and the ability to leverage these data for secondary use (ie, output).

Although patient experience is consistently positively associated with patient safety, clinical effectiveness, and self-rated and objectively measured health outcomes [109], there remains a paucity of empirical research that directly investigates patient experience in digital hospital environments. Our results revealed that, in a small number of studies, patient satisfaction scores were positively associated with digital hospitals (4/61, 7%) or remained unchanged (3/61, 5%). Patients and clinicians shared positive overall impressions of EMRs and negative attitudes toward data privacy in a digital hospital environment; however, this result must be considered in the disproportionate context of patient and clinician experience data reported in studies (10/61, 16% vs 55/61, 90%, respectively).

Patient experience in a digital hospital was sometimes inferred from surrogate measures, including hospital recommendations and discharge information quality, that do not capture the complexity of personal experience. For patients, crude quantitative measurement of satisfaction with digital hospitals neglects the complex nature of the digital patient experience [110] or emerging consumer digital health themes (eg, ethical implications, security, choice, privacy, transparency, accuracy, user-friendliness, and equity of access) [111,112]. The authors support the call by Viitanen et al [23] to develop a framework to describe the different aspects of patient experience and correlate them with appropriate methods for studying patient experience in this context.

The effect of digital hospitals on the clinician-patient relationship was consistently reported by clinicians as a negative outcome of digitization. These results align with the well-researched relationship between EMRs and the clinician-patient dynamic, with evidence supporting negative communication outcomes (eg, rapport, quality of interaction, and time) [113] from a clinician perspective. Patient perceptions of clinician-patient communication when using EMRs are relatively stable in previous systematic reviews despite objective studies describing potentially negative (eg, interrupted speech) and positive (eg, facilitating questions) effects [16]. Patient portals within EMRs can improve clinician-patient communication and should be considered a necessary infrastructure for health services implementing EMRs to mitigate potential negative effects [24].

### Implications for Practice

“Improved patient experience” and “improved clinician experience” are 2 quadrants of the Quadruple Aim of health care that warrant significant health service investment amidst the widespread digital transformation of health care. Our findings highlight the need to address pervasive barriers to positive clinician and patient experiences in digital hospitals. To tackle the issues of clinician-patient interaction, inefficient documentation, workload, and burnout identified in this review, health services can invest in feasible and cost-effective solutions such as clinical education and training that are tailored to each clinical discipline as each discipline has unique EMR needs [10]. Prioritizing investment in patient-facing digital solutions such as patient portals can democratize clinical knowledge and empower patients on their unique health care journey [24].

Investment in optimizing EMR infrastructure will build a strong foundation for new clinical applications in descriptive, predictive (ie, artificial intelligence [AI]), and prescriptive (ie, causal AI and decision support) analytics that can benefit clinical workflows and patient outcomes [114]. COVID-19 initiated the rapid adoption of virtual health care [115], and clinical applications of AI are rapidly emerging as the future primary disruptors of health care [116]. Global health services are building machinery to shift from reactive (treat-manage) to proactive (predict-prevent) models of care, with evidence of success for acute clinical problems such as reducing mortality rate and organ failure in the early identification of sepsis [117]. Stakeholder perspectives on implementing clinical AI have been recently consolidated in a qualitative evidence synthesis [118]; however, similarly to our review, patients, carers, and consumers were an underrepresented group compared with clinicians (11.4% vs 70% of data, respectively).

Our approach of using AI (machine learning) via Leximancer to perform the qualitative evidence synthesis is a novel approach compared with recent manual evidence synthesis methods [118-120]. The use of semiautomated content analysis tools such as Leximancer can accelerate progress toward a learning health system. These tools offer an accelerated pipeline for analyzing “big” qualitative data that suffer from traditional yet burdensome manual analytic workflows. Key health care use cases of applying digital tools to routine analytical workflows are patient-reported experience measures [121], unstructured clinical notes in EMRs [122], and social media [123]. Natural language processing and machine learning have been tested to analyze free-text comments from patient experience feedback [124]. Applications of Leximancer can be pushed by investigating how its algorithms can be used in real-world health care to drive continuous cycles of quality improvement with greater speed and efficiency compared with a manual control. Leximancer offers an impartial starting point for content analysis by automating the identification of key concepts and themes that warrant further qualitative refinement by the research team.

### Limitations

The scope of this review was limited to experiences in a digital hospital environment, and thus, the experiences of specific digital systems (eg, telehealth and patient portals) were not considered. The complex, interacting factors that influence experience; the stage of digital hospital implementation; and the differences among settings were not explored in this review and offer important foci for future research. By not including gray literature and articles not published in English, our search strategy may have missed informal evaluations of clinician and patient experience of digital hospitals (eg, within health service annual reports) and geographical variation in digital hospital evaluations. The heterogeneity of digital health environments is reflected in the heterogeneity of studies included in this review, meaning that it is difficult to draw definitive conclusions agnostic to time and place. One limitation of using Leximancer for the qualitative evidence synthesis is that it does not automatically identify emotive concepts; these are identified by researchers when interpreting the results. Although Leximancer reduces the potential for human bias when compared with manual analysis, researcher interpretation of Leximancer results remains a gateway for introducing bias [40]. A qualitative evidence synthesis for patient experience studies was not possible as we only identified one eligible study. The patient experience results should be interpreted with caution because of the relatively limited patient experience data in studies (10/61, 16%) compared with clinician experience data in studies (55/61, 90%).

### Conclusions

The clinician experience of digital hospitals appeared positive according to high-level indicators (eg, overall satisfaction and data accessibility); however, the qualitative evidence synthesis revealed substantive tensions between digital hospitals and overall experience, such as weakening clinician-patient interaction, change burden, and inefficient data workflows. There is insufficient evidence to draw a definitive conclusion on the patient experience of digital hospitals, but quantitative indications of satisfaction appear positive or agnostic to digitization. Future research must prioritize investigating the patient experience in digital hospitals and measuring the link between exposure (digital hospital) and outcome (experience) in carefully designed pragmatic trials. Areas of interest include examining the interacting factors that influence experience, the stage of digital hospital implementation, and the differences among settings. Equitable investigation of the patient (including pediatric patients) and clinician digital hospital experience must be prioritized in future research. Worldwide, as digital health becomes inseparable from hospitals and general health care, understanding how to optimize the clinician and patient experience in digital hospital environments will be critical to achieving the Quadruple Aim of (digital) health care.

## Appendix

Multimedia Appendix 1 PRISMA (Preferred Reporting Items for Systematic Reviews and Meta-Analyses) 2020 checklist.

Multimedia Appendix 2 ENTREQ (Enhancing the Transparency in Reporting the Synthesis of Qualitative Research) guideline checklist.

Multimedia Appendix 3 Final search strategies for all included databases.

Multimedia Appendix 4 Data extraction template.

Multimedia Appendix 5 Mixed Methods Appraisal Tool results.

Multimedia Appendix 6 Characteristics of the studies included in the systematic review and qualitative evidence synthesis on clinician and patient experience in digital hospitals (N=61).

Multimedia Appendix 7 Leximancer results—qualitative evidence synthesis.

## Abbreviations

AI: artificial intelligence
EMR: electronic medical record
ENTREQ: Enhancing the Transparency in Reporting the Synthesis of Qualitative Research
HCAHPS: Hospital Consumer Assessment of Healthcare Providers and Systems
MMAT: Mixed Methods Appraisal Tool
PRISMA: Preferred Reporting Items for Systematic Reviews and Meta-Analyses
V1: version 1
V2: version 2

## Data Availability

The data supporting the findings of this study are available from the corresponding author (OJC) upon reasonable request.
