# Supplementary material for: The Impact of Digital Hospitals on Patient and Clinician Experience: Systematic Review and Qualitative Evidence Synthesis
Source: J Med Internet Res. 2024 Mar 11;26:e47715. doi: 10.2196/47715 (PMC10964148; doi:10.2196/47715)
Supplement: Multimedia Appendix 3 [file jmir_v26i1e47715_app3.docx]

**Multimedia Appendix 3:** Final search strategy of all included databases (PubMed, Embase, Scopus, CINAHL, PsycInfo)

| Search engine used | Date searched | Keyword search terms |
| --- | --- | --- |
| PubMed | 24-06-2021  22-06-2022 | (("Hospitals"[Mesh] OR hospital* [tiab])) AND (("electronic health records"[MAJR] OR electronic health record*[ti] OR electronic medical record*[ti] OR emr[ti] OR EHR[ti] OR digital hospital*[tiab] OR smart hospital*[tiab] OR digital hospital*[tiab] OR computerized medical record [tiab])) AND (satisfaction[tiab] OR experience[tiab] OR attitude*[tiab] OR perception*[tiab] OR opinion*[tiab] OR behavior*[tiab] OR behaviour*[tiab]) |
| Scopus | 24-06-2021  22-06-2022 | ( TITLE-ABS ( "hospital" ) AND TITLE-ABS ( "electronic AND health AND record" OR "electronic AND medical AND record" OR "emr" OR "ehr" OR "digital AND hospital" OR "computerized AND medical AND record" ) AND TITLE-ABS-KEY ( "attitude*" OR "perception" OR "satisfaction" OR "experience" OR "opinion*" OR "behavior*" OR "behaviour*" ) ) |
| PsycInfo | 24-06-2021  22-06-2022 | ((Any Field: (hospital))) *AND* ((title: (electronic medical record)) *OR* (title: (computerized medical record)) *OR* (title: (electronic health record)) *OR* (title: (EMR)) *OR* (title: (EHR)) *OR* (title: (digital hospital))) *AND* ((Any Field: (attitude)) *OR* (Any Field: (perception)) *OR* (Any Field: (behaviour)) *OR* (Any Field: (behavior)) *OR* (Any Field: (experience)) *OR* (Any Field: (satisfaction)) *OR* (Any Field: (opinion))) |
| Embase | 24-06-2021  22-06-2022 | (hospital:ab,ti) AND ('electronic medical record':ti OR 'electronic health record':ti OR emr:ti OR ehr:ti OR 'digital hospital':ti OR 'computerized medical record':ti OR 'smart hospital':ti) AND ('electronic medical record':ti OR 'electronic health record':ti OR emr:ti OR ehr:ti OR 'digital hospital':ti OR 'computerized medical record':ti OR 'smart hospital':ti) |
| CINAHL | 24-06-2021  22-06-2022 | (‘hospital) AND TI (‘electronic medical record’ OR ‘electronic health record’ OR ‘digital hospital’ OR ‘computerized medical records’ OR ‘EMR’ OR ‘EHR’) AND (‘perception’ OR ‘attitude’ OR ‘behaviour’ OR ‘behavior’ OR ‘experience’ OR ‘satisfaction’ OR ‘opinion’) |
