# Supplementary material for: The Impact of Digital Hospitals on Patient and Clinician Experience: Systematic Review and Qualitative Evidence Synthesis
Source: J Med Internet Res. 2024 Mar 11;26:e47715. doi: 10.2196/47715 (PMC10964148; doi:10.2196/47715)
Supplement: Multimedia Appendix 4 [file jmir_v26i1e47715_app4.docx]

**Multimedia Appendix 4:** Data extraction template

| **Item** | **Instructions for researchers** |
| --- | --- |
| Article reference number | For internal use |
| Study authors (Year) | Report first author then 'et al', report the year published |
| Title | Report the study title |
| Country | What country was the study conducted in? |
| Study design | Qualitative, quantitative, mixed-method |
| Study setting | Where was the study conducted? |
| Data collection | How was the data collected? |
| Data Collection Tool | How was the tool developed? |
| Digitisation of care | Describe the digital hospital (e.g. EMR)  Time since digitisation |
| Participant characteristics | Include total numbers and summary demographics |
| Outcome measure/s | What were the primary and/or secondary outcomes of the study? |
| Theoretical framework | Did the study use a theoretical framework to ground their research? |
| Key findings | What were the key findings in 2-3 dot points? |
| Quantitative Findings | Report qualitative findings |
| Qualitative Findings | Report qualitative findings:  Primary/main themes  Secondary/sub-themes  Diverse/minor/unexpected themes  Participant quotations (verbatim)  Text under 'results', 'findings' or 'conclusions'  Text under 'discussion' |
