# Supplementary material for: The Impact of Digital Hospitals on Patient and Clinician Experience: Systematic Review and Qualitative Evidence Synthesis
Source: J Med Internet Res. 2024 Mar 11;26:e47715. doi: 10.2196/47715 (PMC10964148; doi:10.2196/47715)
Supplement: Multimedia Appendix 5 [file jmir_v26i1e47715_app5.docx]

**Multimedia Appendix 5:** Quality of included studies assessed using the Mixed Methods Appraisal Tool (MMAT) criteria in the current systematic review and meta-synthesis of qualitative studies

| **First author, year** | **Study design** | **MMAT score** | **Comment** |
| --- | --- | --- | --- |
| Aldosari et al. | Quantitative descriptive | ★★★★ | 4.4 not met. |
| Alharthi et al. | Quantitative descriptive | ★★★★ | 4.2 not met. |
| Al-Muhjaini et al. | Quantitative descriptive | ★★★ | 4.1, 4.2 not met. |
| Alobo et al. | Quantitative descriptive | ★★★ | 4.2, 4.4 not met. |
| Alsohime et al. | Quantitative descriptive | ★★★ | 4.2, 4.3 not met. |
| Bani-issa et al. | Quantitative descriptive | ★★★★★ | All criteria met. |
| Bardach et al. | Qualitative | ★★★★ | 1.3 not met. |
| Bossen et al. | Mixed-methods | ★★★ | 5.4, 5.5 not met. |
| Boyer et al. | Qualitative | ★★★★★ | All criteria met. |
| Burgin et al. | Qualitative | ★★★★★ | All criteria met. |
| Burkoski et al. | Qualitative | ★★★★★ | All criteria met. |
| Burridge et al. | Mixed-methods | ★★★★ | 5.3 not met. |
| Burridge et al. | Qualitative | ★★★★★ | All criteria met. |
| Chang et al. | Qualitative | ★★★★★ | All criteria met. |
| Claret et al. | Quantitative descriptive | ★★★ | 4.1, 4.3 not met. |
| De Groot et al. | Quantitative non-randomised | ★★★★★ | All criteria met. |
| Dowding D. | Qualitative | ★★★★★ | All criteria met. |
| Eden et al. | Quantitative descriptive | ★★★★★ | All criteria met. |
| Harmon et al. | Quantitative descriptive | ★★★★★ | All criteria met. |
| Holden R | Qualitative | ★★★★ | 1.4 not met. |
| Hu et al. | Quantitative non-randomised | ★★★★★ | All criteria met. |
| Hung et al. | Mixed-methods | ★★ | 5.3, 5.4, 5.5 not met. |
| Jarvis et al. | Quantitative non-randomised | ★★★★★ | All criteria met. |
| Jung et al. | Qualitative | ★★★★★ | All criteria met. |
| Kaipio et al. | Quantitative descriptive | ★★★★★ | All criteria met. |
| Kaipio et al. | Quantitative descriptive | ★★★★ | 4.4 not met. |
| Kazley et al. | Quantitative non-randomised | ★★★★★ | All criteria met. |
| Kutney-Lee et al. | Quantitative descriptive | ★★★★★ | All criteria met. |
| Kutney-Lee et al. | Quantitative descriptive | ★★★★ | 4.4 not met. |
| Lakabala et al. | Quantitative descriptive | ★★★★ | 4.2 not met. |
| Migdal et al. | Quantitative descriptive | ★★★ | 4.3, 4.5 not met. |
| Moerenhout et al. | Qualitative | ★★★★★ | All criteria met. |
| Pruitt et al. | Qualitative | ★★★★★ | All criteria met. |
| Raddaha A. | Quantitative descriptive | ★★★★★ | All criteria met. |
| Ratanawongsa et al. | Mixed-methods | ★★ | 5.1, 5.3, 5.4 not met. |
| Schenk et al. | Mixed-methods | ★★★ | 5.4, 5.5 not met. |
| Schopf et al. | Quantitative descriptive | ★★★★★ | All criteria met. |
| Schwarz et al. | Quantitative non-randomised | ★★★★★ | All criteria met. |
| Shaker et al. | Quantitative descriptive | ★★★★ | 4.3 not met. |
| Strauss B. | Qualitative | ★★★★★ | All criteria met. |
| Strudwick et al. | Mixed-methods | ★★★★★ | All criteria met. |
| Tajirian et al. | Quantitative non-randomised | ★★★★ | 3.1 not met. |
| Tilahun et al. | Quantitative descriptive | ★★★★★ | All criteria met. |
| Top et al. | Quantitative descriptive | ★★★★★ | All criteria met. |
| Top et al. | Quantitative descriptive | ★★★★★ | All criteria met. |
| Tubaishat A. | Qualitative | ★★★★★ | All criteria met. |
| Tubaishat A. | Quantitative descriptive | ★★★★★ | All criteria met. |
| Williams et al. | Quantitative descriptive | ★★★★★ | All criteria met. |
