# Supplementary material for: The Impact of Digital Hospitals on Patient and Clinician Experience: Systematic Review and Qualitative Evidence Synthesis
Source: J Med Internet Res. 2024 Mar 11;26:e47715. doi: 10.2196/47715 (PMC10964148; doi:10.2196/47715)
Supplement: Multimedia Appendix 6 [file jmir_v26i1e47715_app6.docx]

**Multimedia Appendix 6:** Characteristics of the studies included in the systematic review and qualitative evidence synthesis on clinician and patient experience in digital hospitals (N=61).

| Study characteristics | | Studies, n (%) | References |
| --- | --- | --- | --- |
| **Year of publication** | | | |
|  | 2010 to 2012 | 6 (10) | - Boyer et al [44] - Holden [45] - Al-Mujaini et al [46] - Claret et al [47] - Kazley et al [48] - Top and Gider [49] |
|  | 2013 to 2015 | 12 (20) | - Bossen et al [50] - Jarvis et al [51] - Strauss [52] - Top et al [53] - Alharthi et al [54] - Burgin et al [55] - Lakbala and Dindarloo [56] - Migdal et al [57] - Dowding et al [58] - Harmon et al [59] - Shaker et al [60] - Tilahun and Fritz [61] |
|  | 2016 to 2018 | 11 (18) | - Bani‐Issa et al [62] - Chang et al [63] - Schenk et al [64] - Bardach et al [65] - Kaipio et al [66] - Ratanawongsa et al [67] - Aldosari et al [68] - Burridge et al [69] - Abu Raddaha [70] - Strudwick et al [71] - Tubaishat [72] |
|  | 2019 to 2021 | 24 (39) | - Alsohime et al [73] - Burkoski et al [74] - Hung et al [75] - Kutney-Lee et al [76] - Schopf et al [77] - Tubaishat [78] - Williams et al [79] - Alobo et al [80] - Burridge et al [81] - De Groot et al [82] - Eden et al [83] - Hu et al [84] - Kaipio et al [85] - Moerenhout et al [86] - Schwarz et al [87] - Tajirian et al [88] - Cho et al [89] - Heponiemi et al [90] - Jung et al [91] - Kutney-Lee et al [92] - Lloyd et al [93] - Luyten and Marneffe [94] - Pruitt et al [95] - Tian et al [96] |
|  | 2022 | 8 (13) | - Jedwab et al [97] - Al Otaybi et al [98] - Arikan et al [99] - Chivilgina et al [100] - Czernik et al [101] - Monturo et al [102] - Upadhyay and Hu [103] - Welchen et al [104] |
| **Study design** | | | |
|  | Quantitative descriptive | 31 (51) | - Aldosari et al [68] - Al-Mujaini et al [46] - Alharthi et al [54] - Alobo et al [80] - Al Otaybi et al [98] - Alsohime et al [73] - Arikan et al [99] - Bani‐Issa et al [62] - Cho et al [89] - Claret et al [47] - Eden et al [83] - Harmon et al [59] - Kaipio et al [66,85] - Kutney-Lee et al [76,92] - Lakbala and Dindarloo [56] - Lloyd et al [93] - Luyten and Marneffe [94] - Migdal et al [57] - Monturo et al [102] - Abu Raddaha [70] - Schopf et al [77] - Shaker et al [60] - Tian et al [96] - Tilahun and Fritz [61] - Top and Gider [49] - Top et al [53] - Tubaishat [72] - Welchen et al [104] - Williams et al [79] |
|  | Quantitative nonrandomized | 8 (13) | - De Groot et al [82] - Heponiemi et al [90] - Hu et al [84] - Jarvis et al [51] - Jedwab et al [97] - Kazley et al [48] - Schwarz et al [87] - Tajirian et al [88] |
|  | Qualitative | 15 (25) | - Bardach et al [65] - Boyer et al [44] - Burgin et al [55] - Burkoski et al [74] - Burridge et al [81] - Chang et al [63] - Chivilgina et al [100] - Dowding et al [58] - Holden [45] - Jung et al [91] - Tubaishat [78] - Moerenhout et al [86] - Upadhyay and Hu [103] - Pruitt et al [95] - Strauss [52] |
|  | Mixed methods | 7 (11) | - Bossen et al [50] - Burridge et al [69] - Czernik et al [101] - Hung et al [75] - Ratanawongsa et al [67] - Schenk et al [64] - Strudwick et al [71] |
| **Country** | | | |
|  | Australia | 6 (10) | - Burridge et al [69,81] - Schwarz et al [87] - Eden et al [83] - Jedwab et al [97] - Lloyd et al [93] |
|  | Canada | 4 (7) | - Strudwick et al [71] - Burkoski et al [74] - Tajirian et al [88] - Claret et al [47] |
|  | Finland | 3 (5) | - Kaipio et al [66,85] - Heponiemi et al [90] |
|  | Jordan | 2 (3) | - Tubaishat [72,78] |
|  | Oman | 2 (3) | - Abu Raddaha [70] - Al-Mujaini et al [46] |
|  | Saudi Arabia | 5 (8) | - Aldosari et al [68] - Alharthi et al [54] - Alsohime et al [73] - Shaker et al [60] - Al Otaybi et al [98] |
|  | Taiwan | 2 (3) | - Hung et al [75] - Chang et al [63] |
|  | Turkey | 3 (5) | - Top and Gider [49] - Top et al [53] - Arikan et al [99] |
|  | United States | 21 (34) | - Schenk et al [64] - Czernik et al [101] - Strauss [52] - Moerenhout et al [86] - Pruitt et al [95] - Holden [45] - Dowding et al [58] - Bardach et al [65] - Jung et al [91] - Upadhyay and Hu [103] - Kazley et al [48] - Hu et al [84] - Williams et al [79] - Kutney-Lee et al [76] - Harmon et al [59] - Kutney-Lee et al [92] - Migdal et al [57] - Ratanawongsa et al [67] - Jarvis et al [51] - Monturo et al [102] - Tian et al [96] |
|  | Other | 13 (21) | - Burgin et al [55] (United Kingdom) - Bani‐Issa et al [62] (UAE^a^) - Chivilgina et al [100] (Switzerland) - Cho et al [89] (South Korea) - Schopf et al [77] (Norway) - Alobo et al [80] (Nigeria) - De Groot et al [82] (the Netherlands) - Lakbala and Dindarloo [56] (Iran) - Boyer et al [44] (France) - Tilahun and Fritz [61] (Ethiopia) - Bossen et al [50] (Denmark) - Welchen et al [104] (Brazil) - Luyten and Marneffe [94] (Belgium) |
| **Participants** | | | |
|  | Clinicians | 51 (84) | - Schwarz et al [87] - Bardach et al [65] - Boyer et al [44] - Luyten and Marneffe [94] - Chivilgina et al [100] - Upadhyay and Hu [103] - Bossen et al [50] - Welchen et al [104] - Al Otaybi et al [98] - Lloyd et al [93] - Alobo et al [80] - Heponiemi et al [90] - Tilahun and Fritz [61] - Jung et al [91] - Kaipio et al [85] - Bani‐issa et al [62] - Eden et al [83] - Kutney-Lee et al [76] - Strudwick et al [71] - Aldosari et al [68] - Schenk et al [64] - Abu Raddaha [70] - Tubaishat [72] - Top and Gider [49] - Harmon et al [59] - Dowding et al [58] - Chang et al [63] - Burridge et al [81] - Top et al [53] - De Groot et al [82] - Burkoski et al [74] - Arikan et al [99] - Cho et al [89] - Jedwab et al [97] - Pruitt et al [95] - Czernik et al [101] - Tajirian et al [88] - Williams et al [79] - Tubaishat [78] - Schopf et al [77] - Moerenhout et al [86] - Holden [45] - Al-Mujaini et al [46] - Claret et al [47] - Alharthi et al [54] - Burgin et al [55] - Lakbala and Dindarloo [56] - Shaker et al [60] - Kaipio et al [66] - Alsohime et al [73] - Hung et al [75] |
|  | Patients | 6 (10) | - Hu et al [84] - Strauss [52] - Migdal et al [57] - Kazley et al [48] - Monturo et al [102] - Tian et al [96] |
|  | Clinicians and patients | 4 (7) | - Burridge et al [69] - Kutney-Lee et al [92] - Ratanawongsa et al [67] - Jarvis et al [51] |
