# Supplementary material for: The Impact of Digital Hospitals on Patient and Clinician Experience: Systematic Review and Qualitative Evidence Synthesis
Source: J Med Internet Res. 2024 Mar 11;26:e47715. doi: 10.2196/47715 (PMC10964148; doi:10.2196/47715)
Supplement: Multimedia Appendix 7 [file jmir_v26i1e47715_app7.docx]

**Multimedia Appendix 7: Results of the automated text analytics using Leximancer for the meta-synthesis of qualitative studies**

Leximancer’s machine learning analysis illustrates themes as colored bubbles that are heat-mapped according to their frequency (i.e., ‘importance’), with warmer colors (red, yellow) indicating higher importance and cooler colors (blue, purple) lower importance. Concepts are displayed as dots inside each theme bubble and interconnected across themes. Closer proximity of the theme bubbles or concept dots indicates higher co-occurrence and stronger linkage.


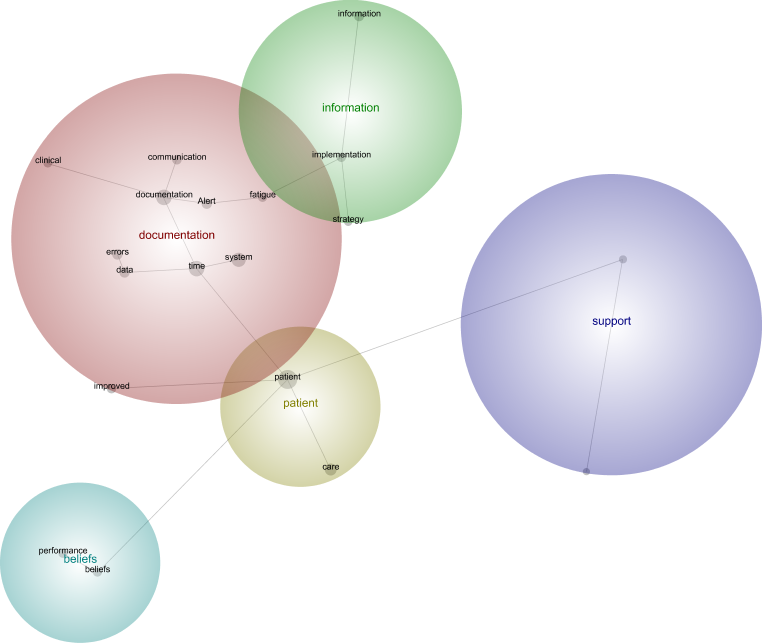


**Figure MA7A: Concept map from Leximancer data analysis – Themes (theme size: 55%; visible concepts: 88%)**


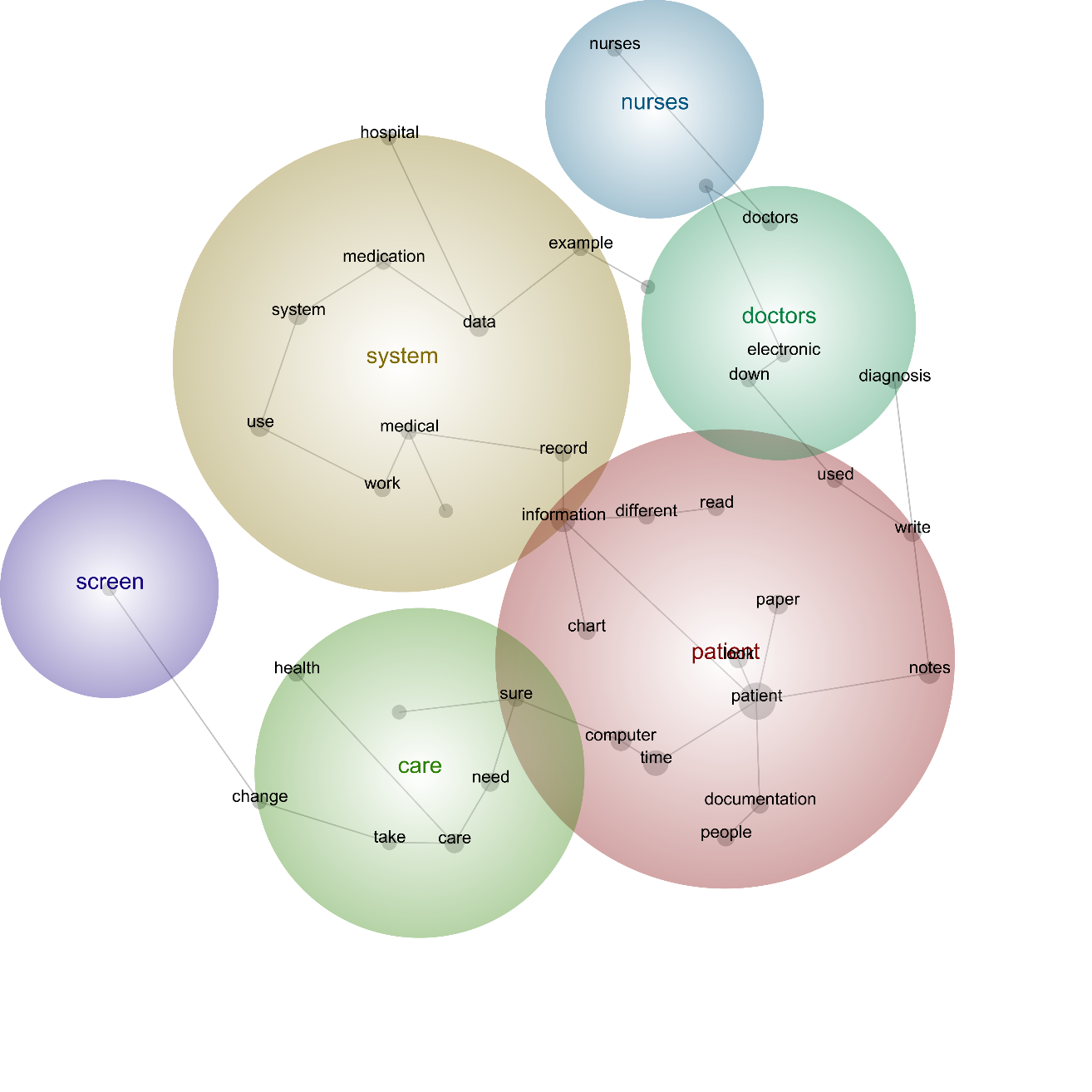


**Figure MA7B: Concept map from Leximancer data analysis – Quotes (theme size: 55%; visible concepts: 88%)**


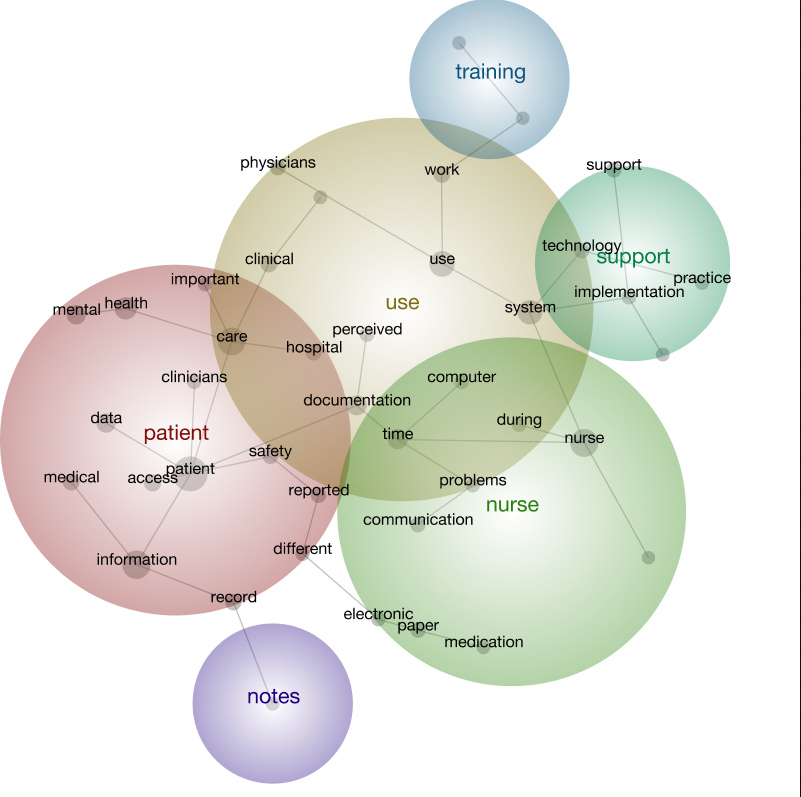


**Figure MA7C: Concept map from Leximancer data analysis – Narrative (theme size: 55%; visible concepts: 88%)**

**Table MA7A: The top five concepts and their two most related concepts identified by Leximancer text analytics**

| **Concepts** | **Themes** | **Quotes** | **Narrative** |
| --- | --- | --- | --- |
| Concept (1) | Patient | Patient | Patient |
| Related Concept (a) | *Documentation* | *Documentation* | *Safety* |
| Related Concept (b) | *Time* | *Read* | *Clinicians* |
| Concept (2) | **Documentation** | **Time** | **Information** |
| Related Concept (a) | *Time* | *Documentation* | *Medical* |
| Related Concept (b) | *System* | *Down* | *Record* |
| Concept (3) | **Time** | **Information** | **Nurse** |
| Related Concept (a) | *Documentation* | *Record* | *Required* |
| Related Concept (b) | *System* | *Wrong* | *Technology* |
| Concept (4) | **Care** | **Notes** | **Use** |
| Related Concept (a) | *Beliefs* | *Read* | *Physicians* |
| Related Concept (b) | *Performance* | *Paper* | *Computer* |
| Concept (5) | **System** | **System** | **Care** |
| Related Concept (a) | *Data* | *Use* | *Mental* |
| Related Concept (b) | *Errors* | *Medication* | *Health* |
